# The influence of sample size and sampling design on estimating population‐level intra specific trait variation (ITV) along environmental gradients

**DOI:** 10.1002/ece3.70250

**Published:** 2024-09-23

**Authors:** Isadora E. Fluck, Sydne Record, Angela Strecker, Phoebe L. Zarnetske, Benjamin Baiser

**Affiliations:** ^1^ School of Natural Resources and Environment University of Florida Gainesville Florida USA; ^2^ Department of Wildlife Ecology and Conservation University of Florida Gainesville Florida USA; ^3^ Department of Wildlife, Fisheries, and Conservation Biology University of Maine Orono Maine USA; ^4^ Institute for Watershed Studies Western Washington University Bellingham Washington USA; ^5^ Department of Environmental Sciences Western Washington University Bellingham Washington USA; ^6^ Department of Integrative Biology Michigan State University East Lansing Michigan USA; ^7^ Ecology, Evolution, and Behavior Program Michigan State University East Lansing Michigan USA

**Keywords:** environmental gradient, intraspecific, ITV, population, trait

## Abstract

Understanding the relationship between intraspecific trait variability (ITV) and its biotic and abiotic drivers is crucial for advancing population and community ecology. Despite its importance, there is a lack of guidance on how to effectively sample ITV and reduce bias in the resulting inferences. In this study, we explored how sample size affects the estimation of population‐level ITV, and how the distribution of sample sizes along an environmental gradient (i.e., sampling design) impacts the probabilities of committing Type I and II errors. We investigated Type I and II error probabilities using four simulated scenarios which varied sampling design and the strength of the ITV‐environment relationships. We also applied simulation scenarios to empirical data on populations of the small mammal, *Peromyscus maniculatus* across gradients of latitude and temperature at sites in the National Ecological Observatory Network (NEON) in the continental United States. We found that larger sample sizes reduce error rates in the estimation of population‐level ITV for both in silico and *Peromyscus maniculatus* populations. Furthermore, the influence of sample size on detecting ITV‐environment relationships depends on how sample sizes and population‐level ITV are distributed along environmental gradients. High correlations between sample size and the environment result in greater Type I error, while weak ITV–environmental gradient relationships showed high Type II error probabilities. Therefore, having large sample sizes that are even across populations is the most robust sampling design for studying ITV‐environment relationships. These findings shed light on the complex interplay among sample size, sampling design, ITV, and environmental gradients.

## Graphical abstract

Linking organismal traits to environmental gradients reveals mechanisms underlying patterns of biodiversity, from individual adaptations to ecosystem‐scale dynamics. This study provides much‐needed guidance for optimizing sampling strategies to improve the accuracy of population‐level characterization of intraspecific trait variability (ITV) and estimation of ITV‐environment relationships, thereby significantly advancing ecological understanding.

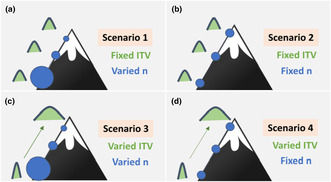

## 1 INTRODUCTION

A central focus of ecology is to understand how species relate to their biotic and abiotic environments. Species traits have long been used as a currency for linking species to environmental gradients (de Bello et al., 2021; McGill et al., 2006) and assessing the influence of biotic interactions on biodiversity patterns (Read, Baiser, et al., 2018; Whittaker, 1972). Traits are commonly used to predict species responses to climatic change and habitat loss (Hoffmann & Sgrò, 2011; MacLean & Beissinger, 2017), assess how assemblages and food webs reorganize following colonization or emigration of new species (e.g., invasion; Baiser et al., 2010; Baiser & Lockwood, 2011; Miehls et al., 2009), and unravel large‐scale patterns in species distributions across geographic gradients (Carvalho et al., 2023; Montaño‐Centellas et al., 2020; Siders et al., 2022).

Although functional traits mediate species‐environment relationships at the individual level, intraspecific trait variation (ITV) has long been neglected in ecological studies. It was only in the past decade that the field of trait‐based ecology experienced a resurgence in the use of individual‐level trait measures to advance population and community ecology (Bolnick et al., 2011; Violle et al., 2012). In contrast to the mean‐trait approach, ITV reflects niche width, providing insights into organism‐environment interactions and the mechanisms driving biodiversity patterns (Fajardo & Siefert, 2019). For example, ITV has been shown to influence coexistence among species (Hogle et al., 2022; Holdridge & Vasseur, 2022; Jordani et al., 2019; Stump et al., 2022), macroecological relationships (Read, Baiser, et al., 2018; Read, Grady, et al., 2018), the establishment of invasive species (Helsen et al., 2020; Westerband et al., 2021), and species distributions across space and time (Fang et al., 2019; Guisan et al., 2019; He et al., 2021; Violle et al., 2012) with implications for community assembly and structure (Lavorel & Garnier, 2002; Siefert et al., 2015; Westerband et al., 2021; Wickman et al., 2022).

Intraspecific trait variation can be studied at a variety of spatial scales (i.e., grains and extents; Wiens, 1989), providing information on the level of biological organization of interest (e.g., populations, communities, ecosystems). For example, in studies that aim to assess patterns of trait variation for a species (e.g., body size variation along latitudinal gradients; Bergmann's Rule, 1847), the spatial extent can be the entire species' geographical range. In such an approach, the spatial grain is often locations of individuals opportunistically collected across the species range (e.g., Guralnick et al., 2020; Riemer et al., 2018). On the opposite end of the spectrum, the spatial extent and grain may be a single location where individuals are exhaustively sampled and measured to capture population‐level ITV from a single population (e.g., Pease & Mattson, 1999). Exhaustive sampling can also be conducted at several locations (e.g., across an environmental gradient) to capture population‐level ITV for multiple populations (e.g., Westerband et al., 2021). Furthermore, population‐level ITV can be calculated for multiple populations of co‐occurring species in a given location to give insight into the trait overlap of coexisting species within a community (Read, Baiser, et al., 2018).

Regardless of the spatial extent or grain or whether an ITV study focuses on one species or an entire community, accurately quantifying ITV is challenging for several reasons. First, estimating ITV hinges on the successful capture and measurement of multiple individuals, which can be constrained by cost, time, and capture rate among other challenges. Second, there is little to no guidance on the number of individuals necessary to accurately estimate ITV. In many studies, researchers set an a priori sample size based on available resources or select *n* = 10 as a sample size (Gotelli & Ellison, 2004). In other studies, ITV is simply calculated based on the number of individuals that end up being sampled (or obtained from museum specimens), which can vary across populations, species, or environmental gradients (e.g., Read, Baiser, et al., 2018). Previous simulations based on in silico populations and empirical data revealed that sample sizes greater than 20 individuals significantly improved the accuracy of population‐level ITV estimation (Yang et al., 2020). However, there are many factors that may influence our conclusions about ITV and its relationship with environmental gradients that have yet to be explored, such as sample size and sample design.

The lack of information on ITV or bias in ITV estimates can limit the understanding of how species respond to environmental changes. Without accurately capturing ITV, scientists may overlook the adaptive potential of individuals, leading to inaccurate predictions of species survival, invasion, adaptation, and ecosystem resilience (Moran et al., 2016). In this study, we explored how sample size affects the estimation of population‐level ITV, and how the distribution of sample sizes along an environmental gradient (i.e., sampling design) affects the inferences we can draw regarding relationships between population‐level ITV and environmental gradients. We sought to understand when different sampling designs along an environmental gradient would lead to the conclusion of a significant ITV–environment relationship when there was none (i.e., Type I error) or to the rejection of an ITV–environment relationship when one existed (i.e., Type II error). We simulated four *Scenarios* where we varied the number of individuals sampled from each population, the sample size‐environmental gradient relationship, the ITV of the populations, and the underlying population ITV‐environmental gradient relationship. We performed these simulations for in silico populations, as well as empirical populations of a small mammal (*Peromyscus maniculatus*). We used our simulations to calculate the probabilities of Type I and II errors associated with different sampling designs to inform future studies of population‐level ITV.

## 2 MATERIALS AND METHODS

We ran three sets of simulations to explore how sample size and sampling design influence the estimation of population‐level ITV and the detection of ITV‐environment relationships. The basis of the first two sets of simulations was a collection of 98 in silico populations, each with 100 individuals. Each population had a log‐normally distributed theoretical trait with a mean of 15, and the standard deviation varied across populations at 98 even intervals between 0.01 and 15. For the third set of simulations, we used empirical data on body size variation of *Peromyscus maniculatus* populations across the continental United States collected by the National Ecological Observatory Network (NEON). For a detailed summary of the simulation parameters, see Table S1.

### 2.1 In silico simulations

#### 2.1.1 1^st^ set of simulations: In silico sample size simulation

In our first simulation, we explored how sample size influenced the estimation of population‐level ITV. From each in silico population described above (*n* = 98), we randomly sampled from 2 to 99 individuals without replacement and measured ITV as the coefficient of variation (standard deviation/mean; *CV*) of the individuals sampled. We denoted this estimate of ITV as $\hat{\mathrm{CV}}$. We repeated this process 1000 times and calculated root mean square error (RMSE) between the 1000 $\hat{\mathrm{CV}}$ values and true population‐ITV (measured as the CV of all 100 individuals in a population) for each sample size–standard deviation combination. Therefore, for this first set of simulations, we gathered a total of 9604 RMSE values, one for each sample size–true population‐level ITV combination.

#### 2.1.2 2^nd^ set of simulations: In silico sample size‐environmental gradient simulations

**FIGURE 1:**
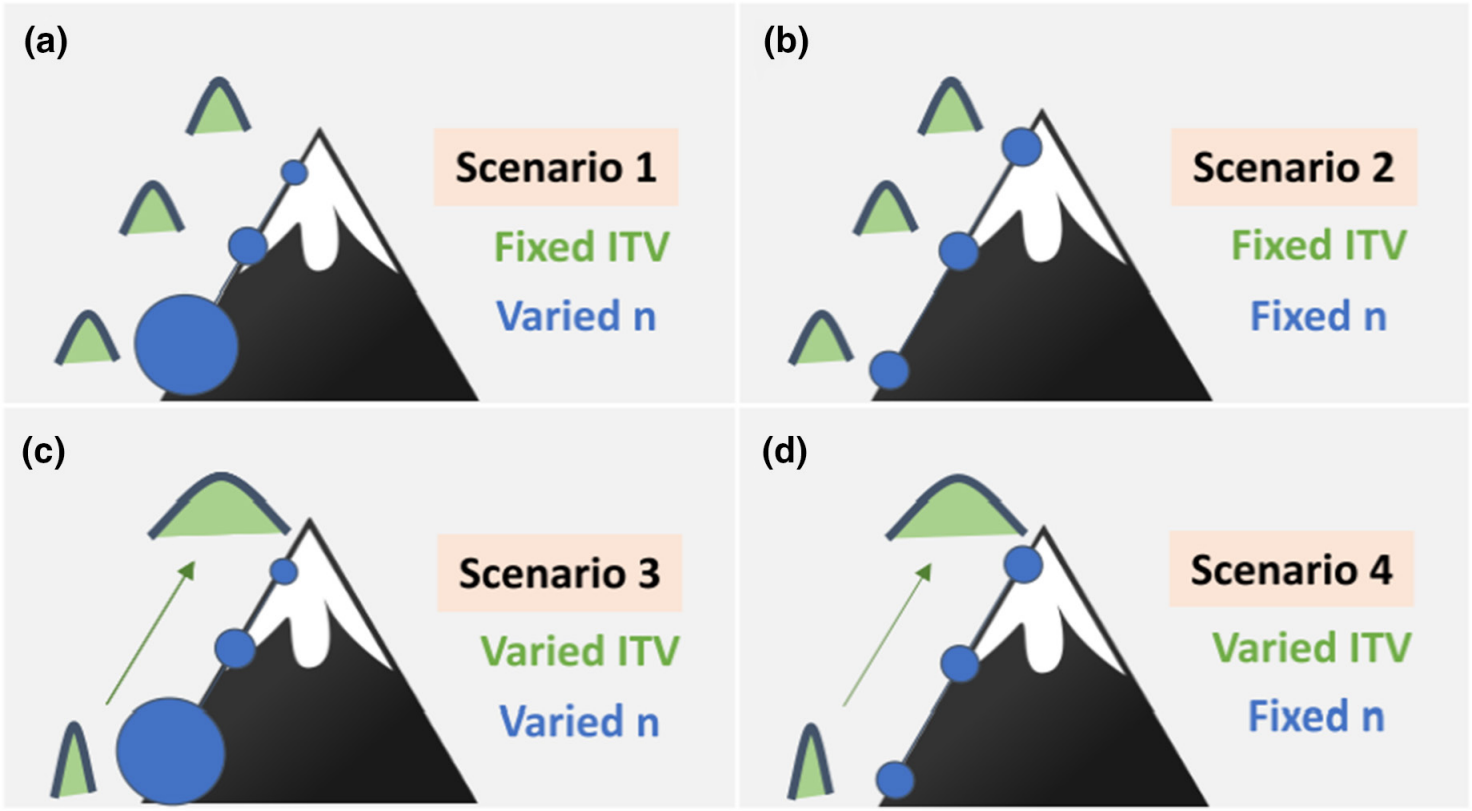
Schematic illustration representing the four simulation *Scenarios* where the mountain (i.e., elevation) represents an environmental gradient, the blue circles represent the sample size and the green parabolas represent the intraspecific trait variation (ITV) of the in silico populations. (a) *Scenario one* represents the case where true population‐level ITV remained the same along the environmental gradient (i.e., fixed ITV), and the researcher varied the number of individuals (n) collected along an environmental gradient (i.e., varied *n*). (b) *Scenario two* represents the case where both ITV and sample sizes are constant along the gradient (i.e., Fixed ITV and Fixed *n*). (c) In *Scenario three*, ITV and sample size varied along the gradient (i.e., Varied ITV and Varied *n*). (d) In *Scenario four*, the true population‐level ITV changed along the environmental gradient (Varied ITV), but the sample size collected from each population was constant (Fixed *n*).

Our second set of simulations assessed ITV for in silico populations evenly arrayed across a theoretical environmental gradient (e.g., elevation for illustration in Figure 1), which was given an arbitrary range of values from 1 to 10 in increments of one. For a set of 10 populations (see also Figures S5 and S6 for simulations with 5 and 15 populations), we simulated relationships between true population‐level ITV, sample size, and an environmental gradient in four different *Scenarios*. In *Scenarios one and two*, true population‐level ITV remained constant along the gradient (Figure 1a,b), whereas in *Scenarios three and four*, true population‐level ITV was significantly correlated with the gradient (Figure 1c,d). Thus, in *Scenarios one and two*, we explored Type I error rates (i.e., false positives) in ITV‐environmental gradient relationships, and in *Scenarios three and four*, we explored Type II error rates (i.e., false negatives). For schematic examples of the simulation process for each scenario, see Figures S1–S4.

##### In silico scenarios one and two

**FIGURE 2:**
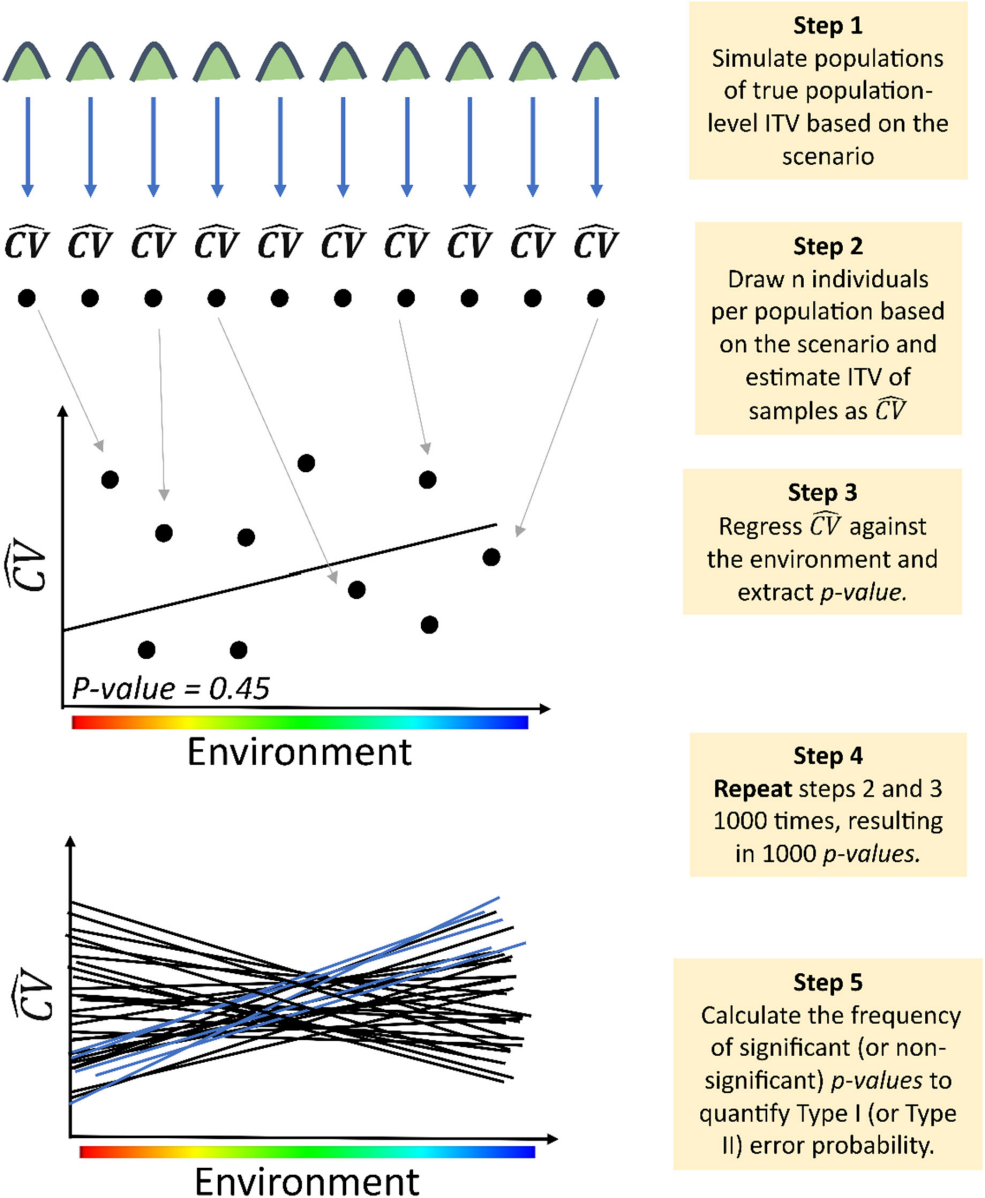
Workflow of the simulation process. After setting the number of individuals to be sampled and the ITV for each population along the gradient according to a given *Scenario*, we followed these steps to assess the probabilities of Type I and II errors. In step one, we define the true population‐level ITV of each population and the relationship to the environmental gradient. In step two, we sampled the number of individuals (n) from each population based on the *Scenario* and estimated ITV as $\hat{\mathrm{CV}}$ for each of the samples. In step three, the $\hat{\mathrm{CV}}s$ calculated in step two become the response variable for a regression against the environmental variable and the *p‐value* of the regression is extracted. In step four, steps one through three are repeated 1000 times to generate 1000 *p‐values*. In step five, we used the frequency of *p‐values* < .05 (or *p‐values* > .05) (blue lines) out of 1000 regressions to calculate the probability of a Type I (or Type II) error.

In *Scenario one* (Figure 1a), there was no relationship between the environmental gradient and true population‐level ITV (i.e., each of the 10 populations in the simulation had identical trait distributions). However, the number of individuals sampled from each population varied and was correlated with the environmental gradient (e.g., capture probability decreased with elevation; Figure 1a). The sample sizes for this *Scenario* ranged from 2 to 99 individuals, with values evenly distributed across the 10 populations (i.e., 2, 13, 24, 34, 45, 56, 67, 77, 88, 99 individuals). We conducted all possible permutations of these 10 values of sample size relative to the gradient, resulting in 3,628,800 arrangements of sample size along the gradient. Then, we randomly selected 100 arrangements that corresponded to sample size‐environmental gradient correlations (*r*) evenly spaced between 0.01 and 1 (Figure S1). For a given sample size–environmental gradient correlation, we randomly sampled the corresponding number of individuals from each population and estimated ITV as the $\hat{\mathrm{CV}}$ of the samples. We then regressed $\hat{\mathrm{CV}}$ against the environmental gradient and extracted the resulting *p‐value*. We repeated this process 1000 times and used the frequency of *p ≤* .05 out of 1000 regressions as a measure of Type I error (see Figure 2, for stepwise schematic). We ran this simulation for all combinations of sample size–environmental gradient correlation (*r*) and population‐level ITV (*n* = 9800).

Like *Scenario one*, in *Scenario two* (Figure 1b), there was no relationship between the environmental gradient and true population‐level ITV (i.e., testing of Type I error). However, the number of individuals sampled from each population was the same across the gradient (i.e., this is a *Scenario* where the researcher sets an equal a priori number of individuals to sample from each population). We tested sample sizes ranging from 2 to 99 for all levels of true population‐level ITV (Figure S2). For a given combination of sample size and true population‐level ITV, we randomly drew the number of individuals from each of the 10 populations and estimated the $\hat{\mathrm{CV}}$ for each sample. We then regressed $\hat{\mathrm{CV}}$ against the environmental gradient and extracted the *p‐value* of the regression. We repeated this process 1000 times and used the frequency of *p* ≤ .05 out of 1000 regressions as a measure of Type I error. We ran this simulation for all combinations of sample size and true population‐level ITV (*n* = 9604).

##### In silico scenarios three and four

In *Scenarios three and four*, we tested the probability of a Type II error. For these two *Scenarios*, true population‐level ITV varied along the environmental gradient (Figure 1c,d). To simulate correlations between ITV and the environmental gradient, we selected 10 populations with true population‐level ITV ranging from SD = 0.01 to 15, which is equivalent to CV = 0.001 to 1 since all the populations were set to have the same mean of 15. The CV values were evenly distributed across the 10 populations (i.e., CV = 0.001, 0.11, 0.23, 0.33, 0.45, 0.54, 0.65, 0.76, 0.86, 1). We conducted all possible permutations of these 10 values of ITV relative to the gradient, resulting in 3,628,800 arrangements of ITV that corresponded to ITV‐environmental gradient correlations (*r*) ranging from 0.01 to 0.99. However, because we wanted to test for a Type II error in this *Scenario* where the alternative hypothesis of true population‐level ITV being correlated with the environmental gradient was true, we filtered correlations for those with *p*‐values ≤ .05, which resulted in 37 correlations that ranged between *r* = .63 and 1. In other words, only 37 arrangements of ITV values along the gradient guaranteed statistically significant correlations between the theoretical environment and true population‐level ITV.

For *Scenario 3* (Figure 1c), the number of individuals sampled from each of the 10 populations was correlated to the environmental gradient (see Scenario one). For a given combination of sample size–environmental gradient and true ITV–environmental gradient correlations, we randomly drew the number of individuals from each of the 10 populations and calculated the $\hat{\mathrm{CV}}$ for each sample (Figure S3). We then regressed $\hat{\mathrm{CV}}$ against the environmental gradient and extracted the *p‐value* of the regression. We repeated this process 1000 times and used the frequency of *p* > .05 out of 1000 regressions to calculate the probability of a Type II error. We ran this simulation for all combinations of correlations between sample size–environmental gradient and true ITV–environmental gradient (*n* = 3700).

In *Scenario four*, there was a relationship between true population‐level ITV and the environmental gradient, but the same number of individuals were sampled for each of the 10 populations (Figure 1d). We created the relationship between true population‐level ITV and the environmental gradient using the methods detailed for *Scenario three* and tested sample sizes ranging from 2 to 99 individuals (Figure S4). For a given combination of sample size and true ITV–environmental gradient correlation, we randomly drew the number of individuals from each of the 10 populations and calculated $\hat{\mathrm{CV}}$ for the samples. Then, we regressed $\hat{\mathrm{CV}}$ against the environmental gradient and extracted the *p‐value* of the regression. We repeated this process 1000 times and used the frequency of *p‐values* > .05 out of 1000 regressions to calculate the probability of a Type II error. We ran this simulation for all combinations of sample size and ITV–gradient correlation (*n* = 3626).

### 2.2 3^rd^ set of simulations: Empirical simulations with *Peromyscus maniculatus* in the United States

To explore how sampling design can influence Type I and II errors with empirical data, we used data from *Peromyscus maniculatus* (deer mice) populations collected by the National Ecological Observatory Network (NEON). NEON covers a wide range of taxa, and its specialized sampling design optimizes sampling practices for each taxonomic group surveyed, requiring different sampling methods to increase detection probability and reduce observer bias. NEON samples small mammal diversity at 47 terrestrial field sites distributed across ecoclimatic domains in the continental US, Hawaii, and Puerto Rico. We gathered data collected from 2013 to 2023 (NEON, 2023a, https://doi.org/10.48443/p4re‐p954; Generated: January 27, 2023). Small mammal sampling plots are 90 m × 90 m (180 m^2^) and consist of a trapping grid of 100 box traps separated by 10 m. Each NEON site has 3–11 small mammal sampling plots. Plots are sampled in 1–3‐night sampling bouts per month depending on weather conditions and site type with a minimum of 4 and a maximum of 6 sampling bouts per year. Each trapped small mammal is identified to species, weighed, sexed, aged, and checked for reproductive status. Full sampling protocols are available in Paull (2022). NEON also collected site‐level environmental variables (NEON, 2023b, version from March 09, 2023; https://www.neonscience.org/field‐sites/explore‐field‐sites).

We selected *P. maniculatus* as a focal species because it is captured in high abundance across many NEON sites (Table S2). Our goal with the *P. maniculatus* data was to simulate the *Scenarios* above on empirical data as a proof‐of‐concept and test the probability of getting Type I and II errors when studying the relationship between ITV in body size and environmental gradients. We selected the 10 sites with the largest numbers of individuals (min = 186, max = 415, mean = 278, and SD = 73) because we wanted our empirical example to be comparable to our simulations and because we needed large sample sizes for each population. We used the coefficient of variation of body weight (g) of all adult individuals sampled at a site as a measure of population‐level ITV.

For *Scenarios one and two* (i.e., testing Type I error), we used latitude as the environmental gradient because it had no significant relationship with population ITV (*R* ^2^ = .018 and *p* = .7098, Figure S7). For *Scenarios three and four* (i.e., testing for Type II error), we were unable to find a significant relationship with any environmental variables for our 10 populations, but we did find a significant relationship between population‐level ITV and mean annual temperature when we removed one site (*R* ^2^ = .462 and *p* = .044, Figure S8). As a result, we used 10 sites for *Scenarios one and two* and nine sites for *Scenarios three and four* (Figures S7 and S8). Since there was a known relationship between true *P. maniculatus* population‐level ITV and the environmental gradients (i.e., no significant correlation with latitude or significant negative correlation with temperature), we did not vary the ITV‐environmental gradient relationship in the empirical simulations.

#### 2.2.1 Empirical sample size simulation

We explored how sample size influenced the estimation of population‐level ITV for 10 populations of *P. maniculatus*. From each population we sampled from 2 to 99 individuals and measured ITV as the coefficient of variation (CV) of the individuals sampled. We denoted this estimate of ITV as $\;\hat{\mathrm{CV}}$. We repeated this process 1000 times and calculated RMSE between the 1000 $\hat{\mathrm{CV}}$ values and true population‐ITV for each sample size.

#### 2.2.2 Empirical sample size‐environmental gradient simulations

##### Empirical scenarios one and two

In empirical *Scenario one*, we determined the number of individuals to sample from each of the 10 populations based on a series of 97 sample size‐latitude correlations (*r*) ranging from 0.01 to 0.97. For each correlation value, we randomly drew the number of individuals from each of the 10 populations and estimated ITV as the $\hat{\mathrm{CV}}$ of each sample. We then regressed $\hat{\mathrm{CV}}$ against latitude and extracted the *p‐value* of the regression. We repeated this 1000 times and used the frequency of *p* ≤ .05 out of 1000 regressions to calculate the probability of a Type I error.

For *Scenario two*, again, the empirical relationship between true population‐level ITV and latitude was not significant (Figure S7 and Table S3). However, in this *Scenario*, the same number of individuals were sampled from each population along the environmental gradient. For each sample size ranging from 2 to 99 individuals, we randomly drew the number of individuals from each of the 10 populations and estimated the $\hat{\mathrm{CV}}$ of the samples. We then regressed $\hat{\mathrm{CV}}$ against latitude and extracted the *p‐value* of the regression. We repeated this 1000 times and used the frequency of *p* ≤ .05 out of 1000 regressions to calculate the probability of a Type I error.

##### Empirical scenarios three and four

In empirical *Scenario three*, there was an observed significant negative relationship between mean annual temperature and true population‐level ITV for the nine populations (Figure S8 and Table S3). We determined the number of individuals to sample from each of the nine populations based on 98 sample size‐temperature correlations (*r*) ranging from 0.01 to 0.98. For each correlation value, we randomly drew the number of individuals from each of the nine populations and estimated the $\hat{\mathrm{CV}}$ of the samples. Then, we regressed the $\hat{\mathrm{CV}}$ against temperature and extracted the *p‐value* of the regression. We repeated this process 1000 times and used the frequency of *p* > .05 out of 1000 regressions to calculate the probability of a Type II error.

Empirical *Scenario four* also had an observed significant negative relationship between temperature and true population‐level ITV for the nine sites (Figure S8 and Table S3). However, like *Scenario two*, the same number of individuals were sampled from each population along the gradient. For each sample size ranging from 2 to 99, we randomly drew the number of individuals from each of the nine populations and estimated ITV of each sample as $\hat{\mathrm{CV}}$. Then, we regressed $\hat{\mathrm{CV}}$ against temperature and extracted the *p‐value* of the regression. We repeated this process 1000 times and used the frequency of *p* > .05 out of 1000 regressions to calculate the probability of a Type II error.

## 3 RESULTS

Our study explored how sample size affects the estimation of population‐level ITV and the inferences we can draw regarding relationships between population‐level ITV and environmental gradients. Overall, we found that increasing sample size reduces error for estimates of ITV, but the influence of sample size on detecting ITV‐environment relationships depends on how sample sizes and true population‐level ITV are distributed along environmental gradients.

### 3.1 In silico simulations

#### 3.1.1 In silico sample size simulation

**FIGURE 3:**
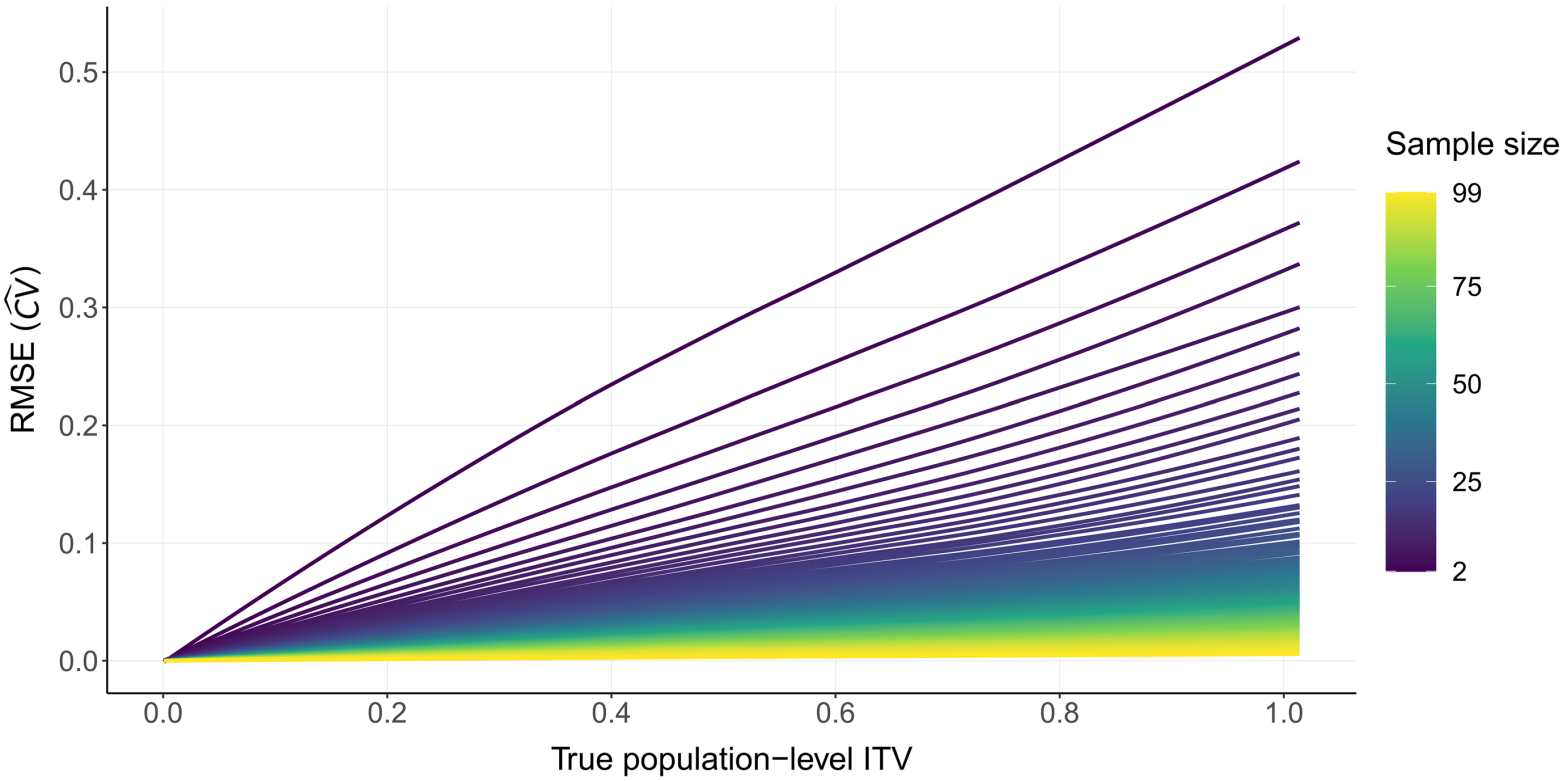
Influence of sample size and true population‐level ITV on the root mean square error (RMSE) of the population‐level ITV estimate ($\hat{\mathrm{CV}}$) for in silico populations.

To explore the role of sample size on the estimation of population‐level ITV, we resampled in silico populations with different levels of ITV across a range of sample sizes. The root mean square error (RMSE) for the estimate of the coefficient of variation ($\hat{\mathrm{CV}}$) for a hypothetical trait increased with population‐level ITV and decreased with larger sample sizes (Figure 3).

#### 3.1.2 In silico sample size‐environmental gradient simulations

##### In silico scenarios one and two

**FIGURE 4:**
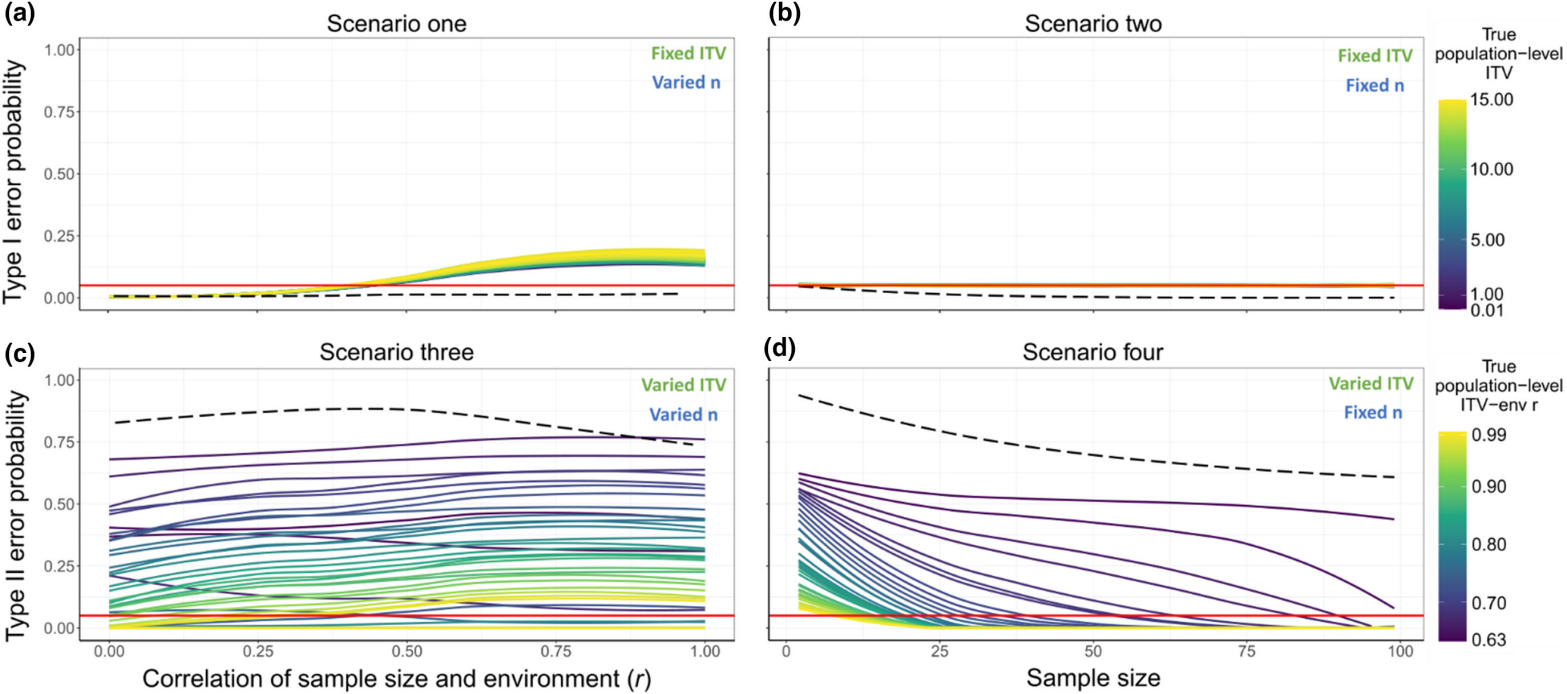
Simulation results across four Scenarios based on in silico and empirical data. Colored lines show the results from simulated in silico populations and dashed black lines represent the results from *Peromyscus maniculatus* empirical populations. The red line indicates a 5% error threshold. Scenarios one (a) and two (b) show Type I error probability across true population‐level ITV for different sample size‐environmental gradient correlations and sample sizes, respectively. Scenarios three (c) and four (d) show Type II error probability across ITV‐environmental gradient relationships for different sample size‐environment correlations and sample sizes, respectively. Note that for *Peromyscus maniculatus* populations (dashed black line) the environmental gradient is latitude in (a) and (b) and temperature in (c) and (d).

The results of *Scenario one* (Figure 4a) showed that Type I error probability increased with the correlation between sample size and the environmental gradient. The Type I error probability crossed the threshold of 0.05 at a correlation of *r* ~ .3 between sample size and the environmental gradient for most values of true population‐level ITV (Figure 4a). There was minimal effect of true population‐level ITV on Type I error probability with more variable populations showing slightly greater Type I error at higher correlations between sample size and the environmental gradient.

*Scenario two* results showed no relationship between either true population‐level ITV or sample size and Type I error probability (Figure 4b). The Type I error probability was ~0.05 for all sample sizes.

##### In silico scenarios three and four

*Scenario three* (Figure 4c) showed that Type II error increased as the relationship between true population‐level ITV and the environmental gradient decreased. However, it was only when the true population‐level ITV was highly correlated (*r >* .8) with the environmental gradient that we observed a Type II error rate under 5%, and this was only in a few instances (Figure 4c). On the other hand, for weaker trait‐environment relationships (*r* < .7), Type II error was always greater than 5%. Note that in our simulations, the lowest significant correlation of population‐level ITV along the environmental gradient was *r =* 0.63. Permutations of true population‐level ITV along the environmental gradient (see methods for *Scenarios three and four*) did not yield significant (*p* ≤ .05) relationships at lower correlations, so they could not be used to test for Type II error.

Our results for *Scenario four* (Figure 4d) showed that Type II error increased as the strength of the true ITV–environmental gradient relationship decreased, similar to *Scenario three*. However, in *Scenario four*, sample size moderated the effect of the true ITV–environment relationship on Type II error. At the lowest sample sizes (e.g., *n* = 2 individuals) Type II error was >5% for even the strongest true ITV–environmental gradient relationships (e.g., *r* = 0.99) whereas larger sample sizes decreased Type II error rates below 5% even for weaker ITV–environmental gradient relationships (i.e., *r* = 0.64). Even with the maximum of 99 individuals sampled, the ITV–environmental gradient relationships of *r* = 0.63 yielded a Type II error probability of 44% (Figure 4d).

We conducted the above simulation with 5 and 15 populations (see Figure S5 and S6 for details). Outcomes were qualitatively similar to the 10 population simulation results. An expected pattern emerged in *Scenarios three* and *four* of increased power (i.e., lower Type II error) with an increasing number of populations.

### 3.2 Empirical simulations: *Peromyscus maniculatus*

Overall, results from the *P. maniculatus* simulations were generally congruent with our theoretical simulations from in silico populations.

#### 3.2.1 Empirical sample size simulation

**FIGURE 5:**
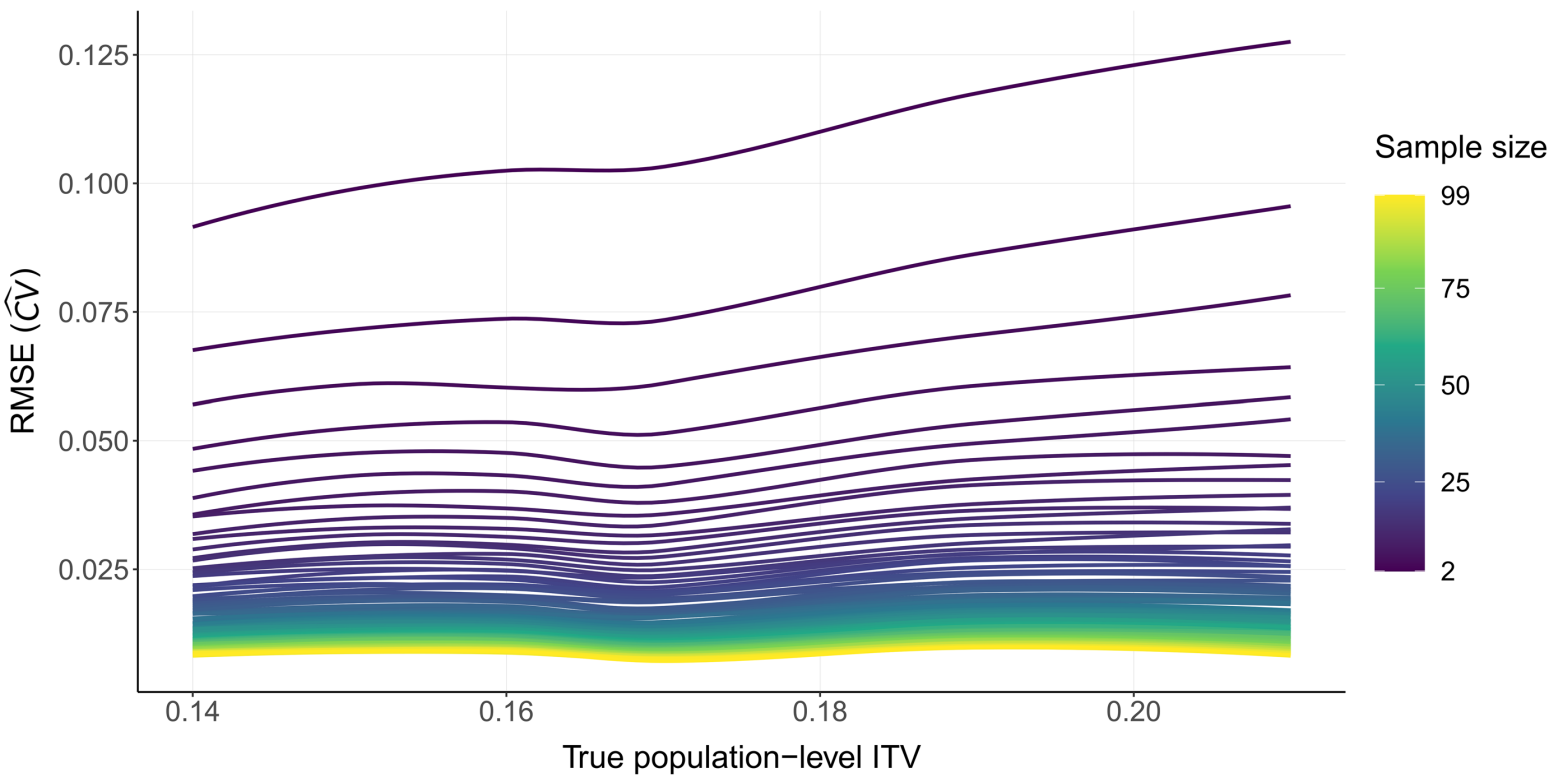
Influence of sample size and true population‐level ITV on the root mean square error (RMSE) of the population‐level ITV estimate ($\hat{\mathrm{CV}}$) for *Peromyscus maniculatu*s populations in the continental United States.

The RMSE of $\hat{\mathrm{CV}}$ for population‐level *P. maniculatus* body size decreased with sample size and slightly increased with population‐level body size ITV (Figure 5). The relationships between RMSE with sample size and population‐level ITV qualitatively matched those of our theoretical simulations (Figure 3) over the same range of true population‐level ITV (i.e., CV = 0.1–0.2).

#### 3.2.2 Empirical sample size‐environmental gradient simulations

##### Empirical scenarios one and two

The results of empirical *Scenario one* showed that Type I error probability was <5% for all sample size–environmental gradient relationships (Figure 4a dashed line). This means that for populations with relatively low ITV, like *Peromyscus maniculatus* (CV ranges from 0.14 to 0.21), the sample size–environmental gradient relationship does not have a strong influence on Type I error probability, especially in the context of an *α* = .05.

In empirical *Scenario two*, there was a decrease in Type I error probability with increasing sample size (Figure 4b dashed line). The lowest sample sizes resulted in Type I errors >5%, but sample sizes greater than 4 individuals consistently resulted in Type I error probabilities <5%. In addition, sample sizes >32 individuals consistently resulted in Type I error probabilities <0.01. Our empirical results deviated from the in silico population simulations, which similarly had low Type I error probabilities but did not decrease with increasing sample size.

##### Empirical scenarios three and four

Empirical *Scenario three* showed that Type II error was highest at low to intermediate sample size–temperature correlations and slightly decreased around a sample size–temperature correlation of *r* = .5 (Figure 4c, dashed line). However, the probability of a Type II error was consistently between 65% and 90%. Thus, regardless of the correlation between the number of individuals sampled from *P. maniculatus* populations and temperature, the observed relationship between body size and latitude was difficult to recover. High Type II error was expected because the observed correlation between ITV and temperature (*r* = .68) was at the lower end of our in silico population simulations (Figure 4c; purple lines).

Empirical *Scenario four* showed that the probability of a Type II error decreased with sample size (Figure 4d dashed line), which matched the results from the in silico population simulations (Figure 4d). The lowest sample size of 2 individuals had Type II error rates of 93% and error rates declined to 60% when a maximum of 99 individuals were sampled per population.

## 4 DISCUSSION

The study of intraspecific trait variation (ITV) requires the successful capture and measurement of multiple individuals, which can be costly in terms of time and money at best, and nearly impossible due to low capture rates among other logistical challenges at worst. As ITV studies within populations and communities from local to macroecological scales have grown over the past decade, little to no guidance has emerged for how many individuals are necessary to accurately estimate ITV (but see Yang et al., 2020). Here, we show that larger sample sizes reduce error rates in the estimation of population‐level ITV for both in silico populations and populations of *Peromyscus maniculatus*. While this result is intuitive, we further show that the influence of sample size on detecting ITV‐environment relationships depends on how sample sizes and true population‐level ITV are distributed along environmental gradients. High correlations between sample size and the environment result in greater Type I error, while weak ITV–environmental gradient relationships are difficult to detect (i.e., greater Type II error) but larger sample sizes help reduce this error.

Simulations based on in silico populations and empirical data have already proven to be a fruitful path to advance methods for quantifying ITV. Yang et al. (2020) found that sample sizes >20 individuals significantly improved the accuracy of ITV estimation. Our estimates of sample size for individual in silico and *P. maniculatus* populations qualitatively agree with Yang et al. (2020). When fewer than ~20 individuals are sampled, there is a rapid increase in error for the estimation of population‐level ITV regardless of the underlying true population ITV. However, the study of population‐level ITV is often extended from one to multiple populations (Costa‐Pereira et al., 2018; Evangelista et al., 2019; Musseau et al., 2020; Stinson et al., 2018; Westerband et al., 2021; Yan et al., 2018) which in many cases, are distributed across environmental gradients to answer the question: Are populations more (or less) variable under certain conditions (Helsen et al., 2017)? We show that the influence of sample size on estimating population‐level ITV is not straightforward in this context.

Based on our simulations, the detection of ITV–environmental gradient relationships can be influenced by: (1) the underlying relationship between true population‐level ITV and the environmental gradient; and (2) the relationship between sample size and the environmental gradient. When there is no underlying ITV–environmental gradient relationships (i.e., *Scenarios one and two*; Figure 1a,b), it is better to have no relationship between sample size and the environment (e.g., most easily achieved by sampling the same number of individuals from each population across the gradient) regardless of sample size and true population‐level ITV. When there is a correlation between sample size and the environment where *r* > ~0.3, Type I error exceeds 5%. On the other hand, when systems do have an underlying population‐level ITV–environmental gradient relationship (i.e., *Scenarios 3 and 4*, Figure 1c,d), weak gradients are difficult to detect and increased sample size can reduce Type II error.

What information do our simulations yield regarding sampling design for ITV researchers? The first challenge in answering this question is that in most cases, there is no a priori knowledge of the strength of the relationship between ITV–environmental gradient (i.e., in most cases, that is why the study is being conducted in the same first place). However, it is clear that the component we can control—the sample size‐environment relationship—should not show a high correlation. If a new experiment is being designed, researchers should plan on sampling the same number of individuals from each population along the gradient. If data have already been collected (e.g., museum specimens or previous studies) the correlation between sample size and gradients of interest should be assessed. If there is a moderate to strong relationship (*r* > .4), samples should be rarified to the sample with the fewest individuals. The second recommendation garnered from our simulations, which is intuitive, is that both sample sizes and the number of populations should be as large as possible to reduce Type II error.

Interestingly, while greater true population‐level ITV increases error in the estimate of ITV for individual populations (Figures 3 and 5), it has little effect on Type I error probability when considering multiple populations across a gradient (Figure 4a,b). Regardless of whether populations are extremely variable for a given trait or if they show almost no ITV, sample size and sample size‐environment relationship don't exert a strong influence on Type I error.

Our results show that some knowledge of the exact elements that are under investigation in an ITV‐environment study is necessary to avoid Type I and II errors. Namely, the underlying population‐level ITV and its relationship with environmental gradients. One way to solve this conundrum is to leverage known information on traits, taxa, and environmental gradients. For example, if ITV‐environment relationships are expected to be strong for a given trait and taxonomic group based on natural history or previous studies (e.g., positive influence of temperature on height ITV of herbs; Helsen et al., 2017; Kuppler et al., 2020; Lemke et al., 2012, 2015), fewer individuals per population would be needed to detect that relationship than if ITV‐environment relationships were generally expected to be weak. Collection of this information from previous and future studies will build knowledge to guide sampling design and sample size for population‐level ITV studies for different taxa, traits, and environmental gradients.

Our empirical example using *P. maniculatus* population‐level ITV for body size across latitudinal and temperature gradients in the continental United States confirmed many of the findings from the in silico population simulations. For the relationship between body size ITV and latitude, we found low rates of Type I error (<5%) regardless of the sample size or sample size‐environment correlation. However, at higher sample size‐environment correlations, we noticed a slight increase in the Type I error rate, although not as large as the increase seen in the in silico populations. The decrease in Type I error rate for *P. maniculatus* with sample size in *Scenario two* differs from the flat line observed for the in silico populations. This is likely due to the natural complexity of the empirical data: although the underlying relationship between true population‐level ITV and the environmental gradient was not significant in both cases, the in silico populations had the same abundance, mean and variance while these parameters varied for *P. maniculatus* populations (but not in relation to latitude). We conclude that the sampling designs represented in empirical scenarios one and two, where we tested the relationship between *P. maniculatus* population‐level body size ITV and latitude, are robust to Type I errors even under sample sizes as low as 5 individuals.

However, such small sample sizes are not enough to avoid Type II error when assessing the relationship between *P. maniculatus* population‐level ITV for body size and temperature. In fact, Type II error never dropped below 50% for any sample size or sample size‐environment correlation for the range of sampling designs in *Scenarios three* and *four*. In other words, the negative relationship between *P. maniculatus* population‐level body size ITV and temperature was detected less than 50% of the time. This study was underpowered and would require a larger number of populations to detect the ITV‐gradient relationship of *r* = 0.68 which is consistent with the results from our in silico population simulations for *Scenarios three and four*.

The best practices that emerge from this study are as follows: (1) While it may seem self‐evident, having large sample sizes of individuals and populations is important for increasing the power to detect ITV‐environmental gradients. (2) Avoid sampling designs where sample size is correlated with the environmental gradient. In cases where data have already been collected and sample sizes are random across populations, we recommend examining the correlation between sample size and environment. If this correlation is found to be strong, its effects can be minimized by rarefying down to the sample size of the population with the fewest individuals. If the results from the rarefied data are similar to those found using the full dataset, researchers can be more confident that the results are not biased by differences in sample sizes across populations.

While we address sample size and design for population‐level ITV, ITV studies often address questions about trait variation at the scale of a species' range (e.g., Guralnick et al., 2020; Riemer et al., 2018), between closely related species (e.g., Read, Grady, et al., 2018) and, at the community scale (Mungee & Athreya, 2021; Siefert et al., 2015; Violle et al., 2012). Expanding the scope of future studies to include multiple species and multivariate environmental gradients in empirical or simulation contexts would provide insights into broader ecological patterns and evolutionary dynamics. However, determining how many individuals are necessary to quantify ITV in a range‐wide or community context likely consists of a different set of challenges and sampling design considerations. Applying what we have learned in our study to other taxa or multiple species in communities arrayed along a gradient may work in some instances, but in others, species may have vastly different abundances (e.g., common or rare), capture probabilities and different underlying ITV‐environmental gradient relationships. Furthermore, ITV‐environmental gradient relationships may also depend on the trait or number of traits selected for analysis. For example, traits associated with movement may have different environmental responses than traits associated with foraging strategy (Dalmolin et al., 2020). Thus, considering multiple traits and their covariation may provide a more detailed understanding of how sample size and sampling design influence Type I and Type II errors. Further simulations are likely needed to gain insight into sampling design for range‐wide and community‐level ITV as well as ITV derived from multiple traits.

Our study advances our understanding of “How many individuals do you need to quantify ITV?”. Unfortunately, there is no golden number. For single populations, our simulations concur with those of Yang et al. (2020) that ~20 individuals can reduce error in the estimate ITV as measured by CV. We show that when estimating ITV‐environmental gradient relationships for multiple populations, the relationship between sample size and the environmental gradient, and the nature of the relationship between underlying true population‐level ITV and the environment must be considered in the sampling design to avoid Type I and II errors.

## AUTHOR CONTRIBUTIONS

**Isadora E. Fluck:** Conceptualization (lead); data curation (lead); formal analysis (lead); investigation (lead); methodology (lead); validation (equal); visualization (lead); writing – original draft (lead). **Sydne Record:** Conceptualization (supporting); data curation (supporting); formal analysis (supporting); funding acquisition (equal); investigation (supporting); methodology (supporting); supervision (supporting). **Angela Strecker:** Conceptualization (supporting); data curation (supporting); formal analysis (supporting); funding acquisition (equal); investigation (supporting); methodology (supporting). **Phoebe L. Zarnetske:** Conceptualization (supporting); data curation (supporting); formal analysis (supporting); funding acquisition (equal); investigation (supporting); methodology (supporting). **Benjamin Baiser:** Conceptualization (equal); data curation (supporting); formal analysis (supporting); funding acquisition (equal); investigation (supporting); methodology (equal); supervision (lead); validation (equal); visualization (supporting); writing – original draft (supporting).

## CONFLICT OF INTEREST STATEMENT

The authors declare no conflict of interest.

## STATEMENT OF INCLUSION

Our study synthesizes local data from multiple regions across the vast expanse of the United States. We emphasize the inclusive nature of our study and ensure a comprehensive consideration of different perspectives by collaborating with authors from several U.S. states and one other country (Brazil), who were actively involved in the research and study design from the beginning. While our study focuses primarily on a simulation with a case study to demonstrate our proposed approach, it inherently encompasses various biological, ecological, and statistical viewpoints and enriches the discourse within functional ecology.

## Supporting information


Data S1.

## Data Availability

All data sources utilized in this research are cited in‐text and in the references section. All data are publicly available in a permanent FigShare repository (https://doi.org/10.6084/m9.figshare.24849444.v1).
