# Supplementary material for: The influence of sample size and sampling design on estimating population‐level intra specific trait variation (ITV) along environmental gradients
Source: Ecol Evol. 2024 Sep 23;14(9):e70250. doi: 10.1002/ece3.70250 (PMC11420108; doi:10.1002/ece3.70250)
Supplement: Supplementary file 1 — Data S1. [file ECE3-14-e70250-s001.docx]

**Supplementary material**


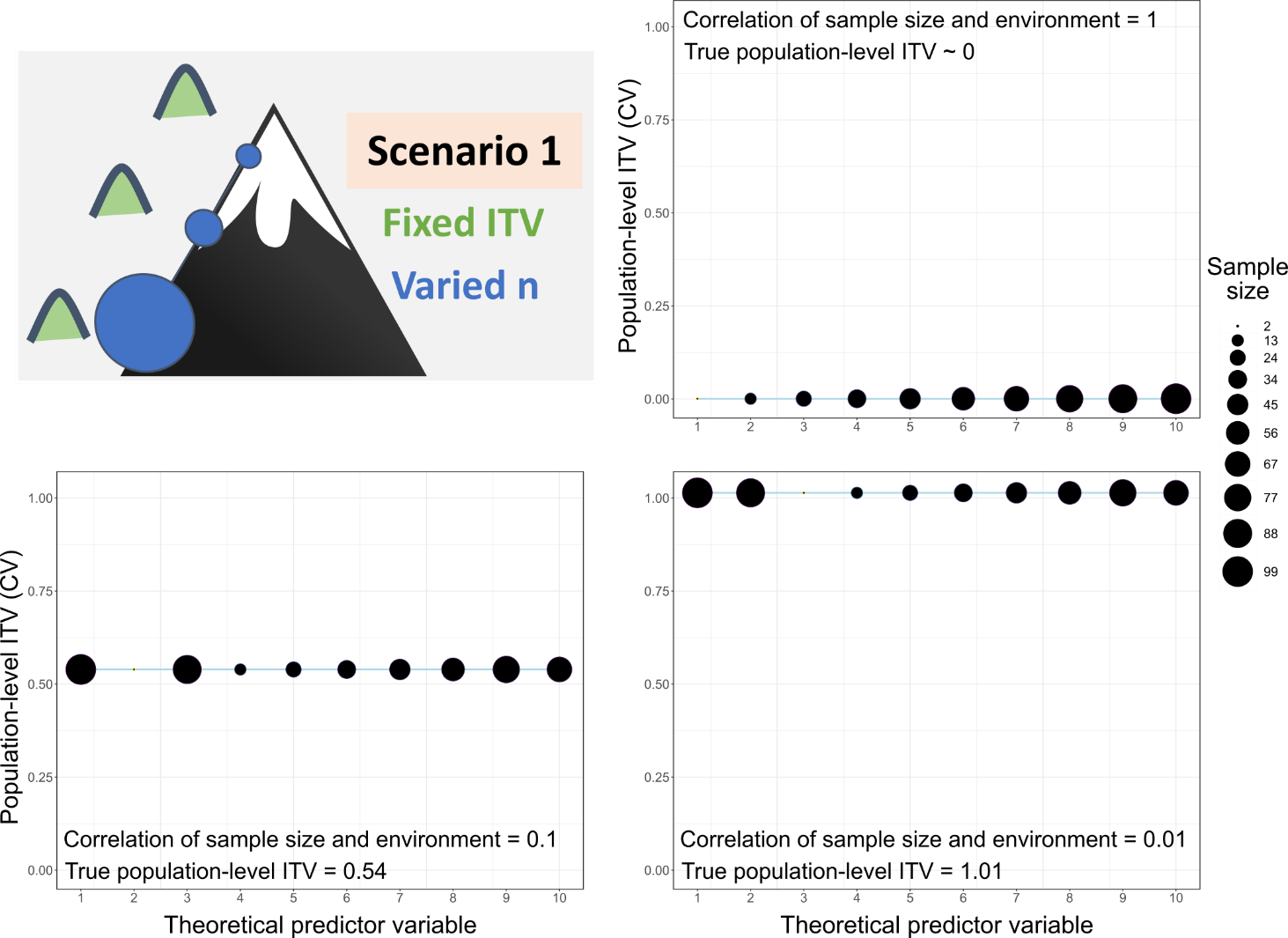


**Figure S1** - Three examples of simulations from Scenario 1, each representing a different combination of (i) correlation between sample size and the theoretical environment and (ii) true population-level intraspecific trait variability (ITV), as indicated by text within each plot. The theoretical environmental variable is represented on the x-axis, and the y-axis represents the population-level ITV (CV). Circle size represents the sample size (n) to be sampled from each population. Sample sizes range from 2 to 99 individuals and are evenly distributed across 10 populations (i.e., 2, 13, 24, 34, 45, 56, 67, 77, 88, and 99 individuals). In this scenario, sample sizes vary across populations, while population-level ITV remains constant across the 10 populations along the theoretical environmental gradient. The flat blue line represents this lack of relationship between population-level ITV and environment. We tested 100 sample size-environment correlations ranging from 0.01 to 1, but here we show examples of sample size permutations that resulted in sample size-environment correlations of 1, 0.1, and 0.01. Similarly, we tested 98 levels of population-level ITV ranging from ~0 to 1.01, but here we show examples of population-level ITV of ~0, 0.54, and 1.01. During the simulation process, we randomly sampled the respective number of individuals from each population and estimated the ITV of the sample as the coefficient of variation ($\hat{\boldsymbol{CV}}$). A regression analysis was then performed between the $\hat{\boldsymbol{CV}}$ estimates and the theoretical environmental gradient. We repeated this process 1000 times to determine the probability of Type I error.


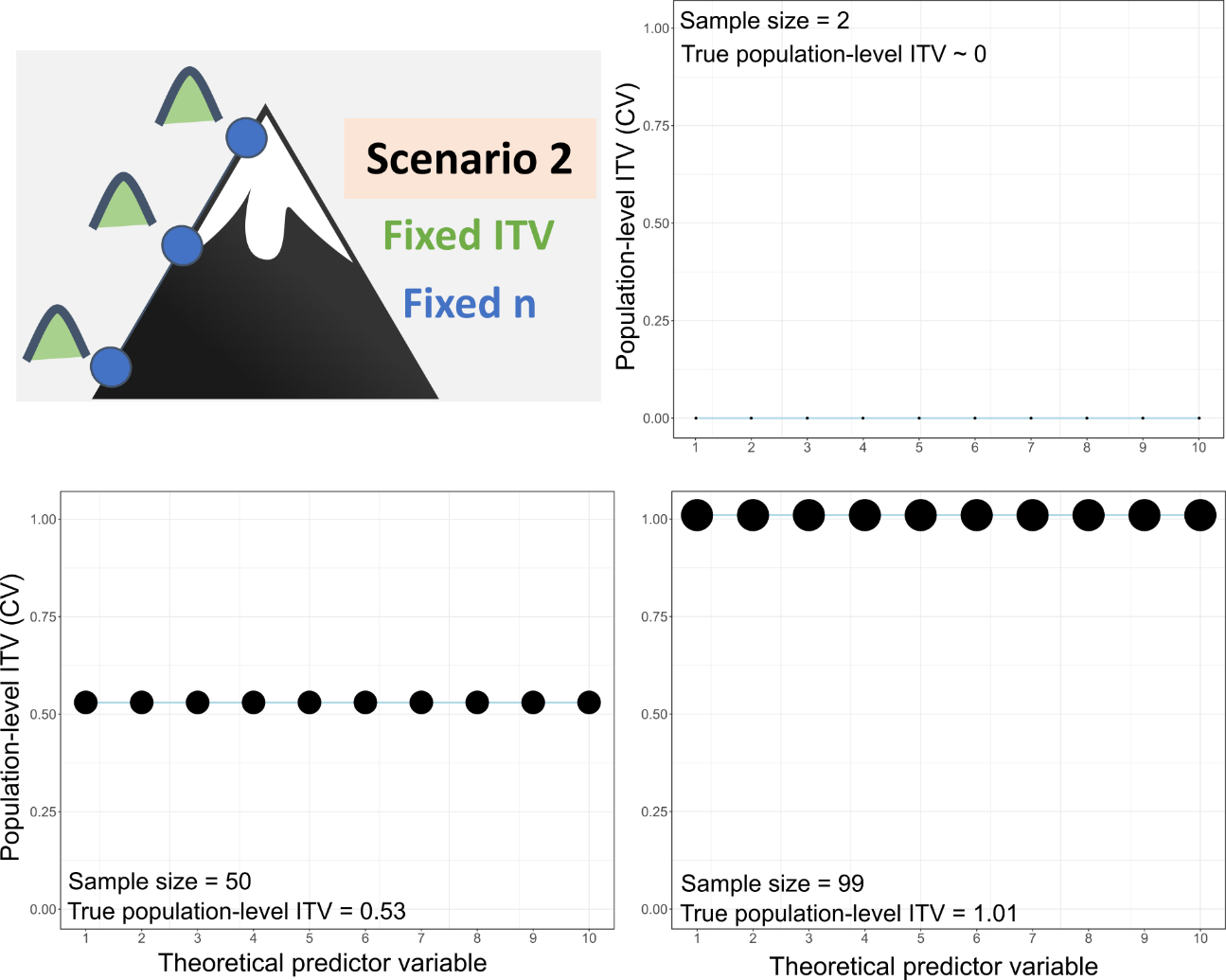


**Figure S2 -** Three examples of simulations from Scenario 2, each representing a different combination of (i) sample size and (ii) true population-level intraspecific trait variability ITV, as indicated by text within each plot. The theoretical environmental variable is represented on the x-axis, and the y-axis represents the population-level ITV (CV). In this scenario, sample sizes and population-level ITV are constant across the 10 populations along the theoretical environmental gradient. The flat blue line represents this lack of relationship between population-level ITV and environment. We tested all of the values for sample sizes from 2 to 99 (total of 98 values), but here we show examples for sample sizes of 2, 50, and 99. Similarly, we tested 98 levels of population-level ITV ranging from ~0 to 1.01, but here we show examples of population-level ITV of ~0, 0.54, and 1.01. During the simulation process, we randomly sampled the respective number of individuals from each population and estimated the ITV of the sample as the coefficient of variation ($\hat{\boldsymbol{CV}}$). A regression analysis was then performed between the $\hat{\boldsymbol{CV}}$ estimates and the theoretical environmental gradient. We repeated this process 1000 times to determine the probability of Type I error.


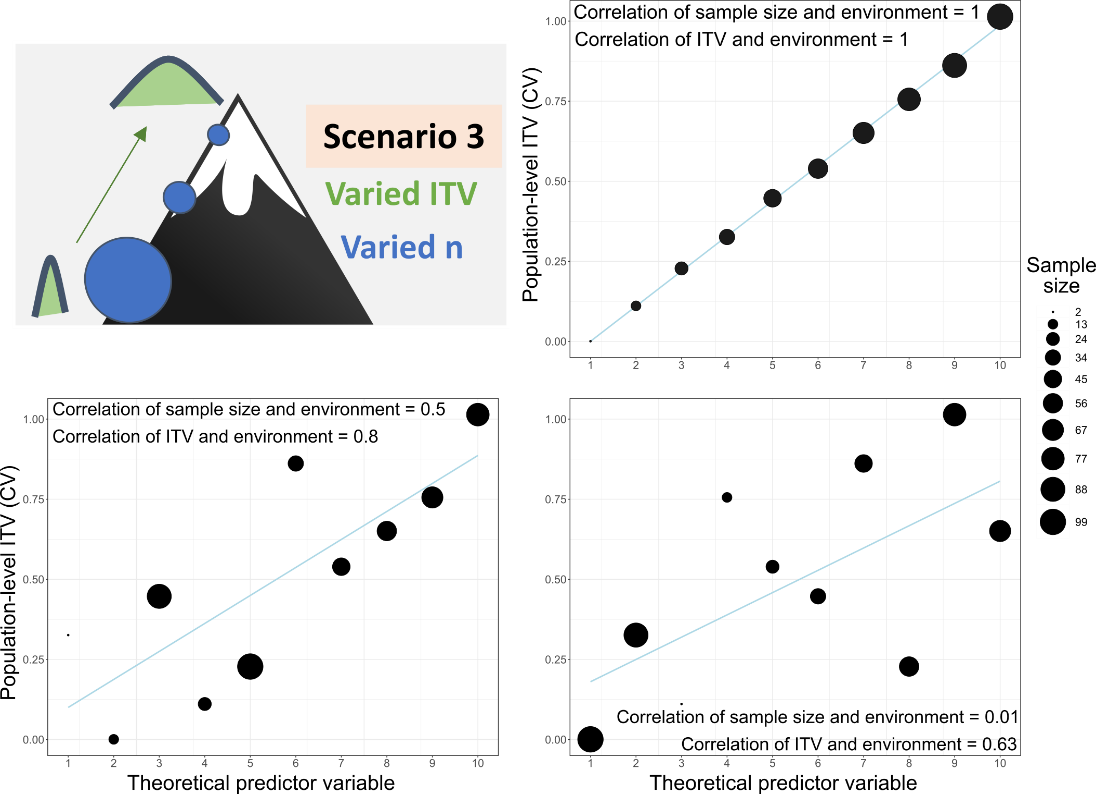


**Figure S3** - Three examples of simulations from Scenario 3, each representing a different combination of (i) correlation between sample size and the theoretical environment and (ii) relationship between population-level intraspecific trait variability (ITV) and theoretical environment, as indicated by text within each plot. The theoretical environmental variable is represented on the x-axis, and the y-axis represents the population-level ITV (CV). The CV values range from 0.01 to 1 and are evenly distributed across the 10 populations (i.e., CV = 0.001, 0.11, 0.23, 0.33, 0.45, 0.54, 0.65, 0.76, 0.86, 1). Circle sizes represent the sample size to be sampled from each population. Sample sizes range from 2 to 99 individuals and are evenly distributed across 10 populations (i.e., 2, 13, 24, 34, 45, 56, 67, 77, 88, and 99 individuals). In this scenario, both sample sizes and population-level ITV vary across the 10 populations along the theoretical environmental variable. We tested 100 correlations between sample size and environment ranging from 0.01 to 1, but here we show examples of sample size permutations that resulted in sample size-environment correlations of 1, 0.1, and 0.01. Similarly, we tested a total of 37 significant population-level intraspecific trait variability (ITV)-environment relationships ranging from 0.63 to 1, but here we show examples of relationships of r=1, 0.8, and 0.63. The blue line represents these relationships between population-level ITV and environment. During the simulation process, we randomly sampled the respective number of individuals from each population and estimated the ITV of the sample as the coefficient of variation ($\hat{\boldsymbol{CV}}$). A regression analysis was then performed between the $\hat{\boldsymbol{CV}}$ estimates and the theoretical environmental gradient. We repeated this process 1000 times to determine the probability of Type II error.


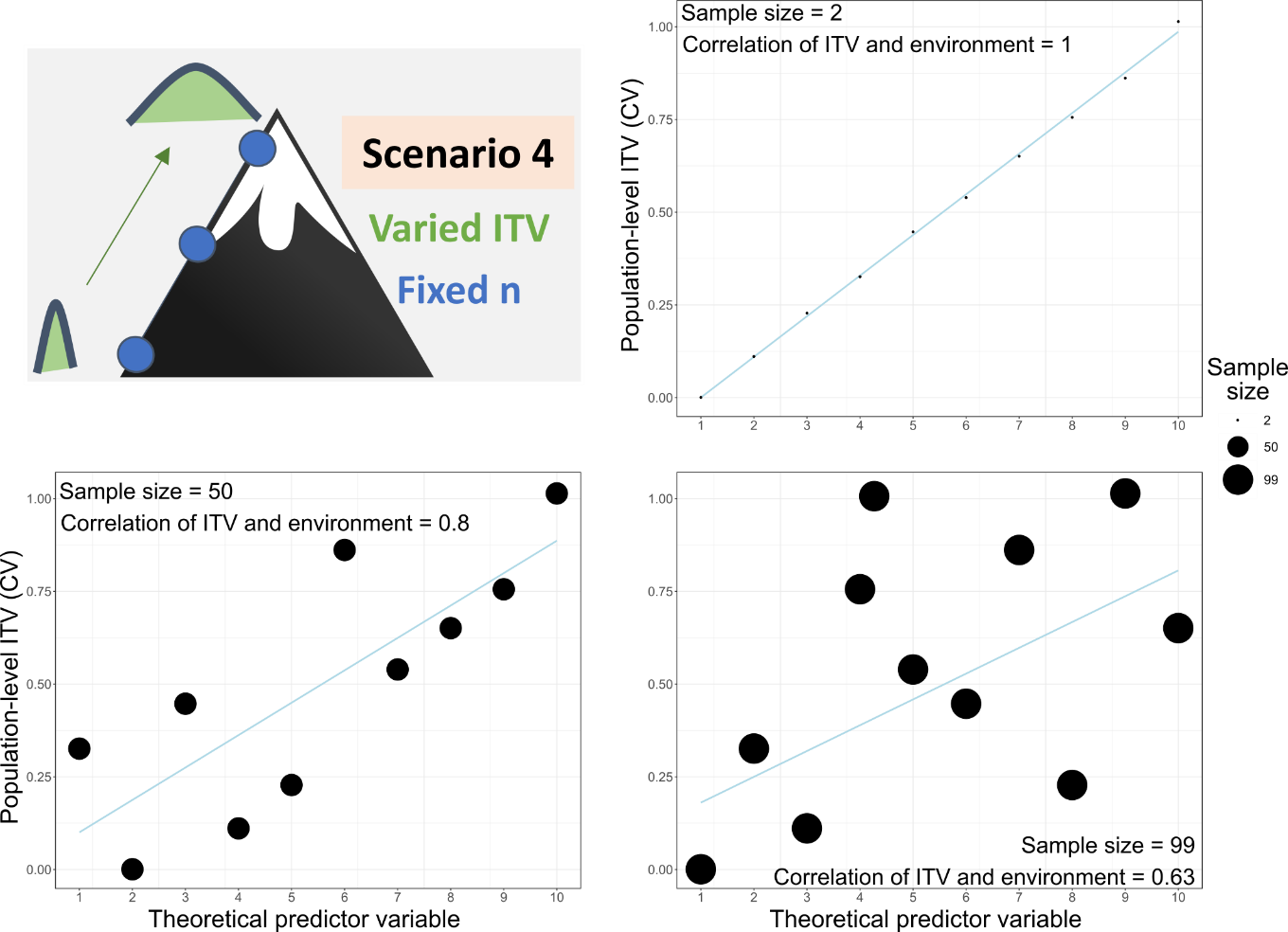


**Figure S4** - Three examples of simulations from Scenario 4, each representing a different combination of (i) sample size and (ii) relationship between population-level intraspecific trait variability (ITV) and theoretical environment, as indicated by text within each plot. The theoretical environmental variable is represented on the x-axis, and the y-axis represents the population-level ITV (CV). The CV values range from 0.01 to 1 and are evenly distributed across the 10 populations (i.e., CV = 0.001, 0.11, 0.23, 0.33, 0.45, 0.54, 0.65, 0.76, 0.86, 1). In this scenario, sample sizes remain constant across the populations, while population-level ITV vary across the 10 populations along the theoretical environmental variable. We tested all of the values for sample sizes from 2 to 99 (total of 98 values), but here we show examples for sample sizes of 2, 50, and 99. Similarly, we tested a total of 37 significant population-level intraspecific trait variability (ITV)-environment relationships ranging from 0.63 to 1, but here we show examples of relationships of r=1, 0.8, and 0.63. The blue line represents these relationships between population-level ITV and environment. During the simulation process, we randomly sampled the respective number of individuals from each population and estimated the ITV of the sample as the coefficient of variation ($\hat{\boldsymbol{CV}}$). A regression analysis was then performed between the $\hat{\boldsymbol{CV}}$ estimates and the theoretical environmental gradient. We repeated this process 1000 times to determine the probability of Type II error.


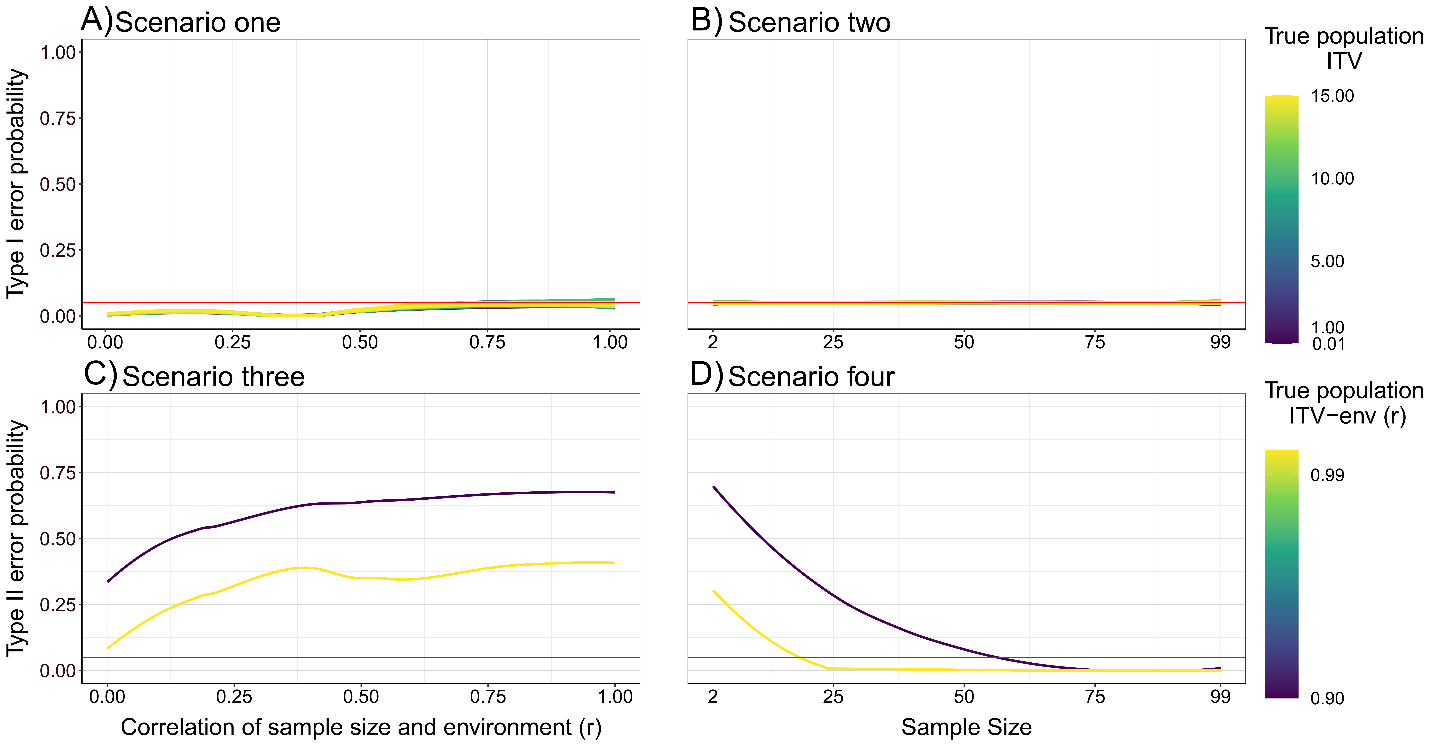


**Figure S5**. *In silico* population simulations for 5 populations. Five populations were evenly distributed across the same environmental gradient ranging from 1 to 10 (1, 3.25, 5.50, 7.75, 10). For *Scenarios* which ITV varied across populations, we used population-level ITV values of 0.01, 3.72, 7.43, 11.29 and 15, and for *Scenarios* which sample size varied along the environment, we used sample sizes of 2, 26, 50, 75 and 99. When ITV and sample size were fixed across populations, 98 values of standard deviation ranged from 0.01 to 15 and sample sizes ranged from 2 to 99. **A)** Findings from *Scenario 1* followed the trend of the results using 10 populations, which is higher probability of Type I error with increasing correlation between sample size and environment. However, the correlation between sample size and environment exhibited diminished importance for Type I error probabilities when using 5 populations. On the other hand, *Scenario 2* **B)** results demonstrated no effect of sample size on Type I error probability. More specifically, Type I error probabilities remained constant at the level of 0.05 across different sample sizes, which corroborates with the results for 10 populations. For Type II error probabilities, only ITV-environment correlations of 0.90 and 0.99 were found to be significant (p ≤ 0.05) and thus, applicable in *Scenarios 3 and 4*. **C)** In *Scenario 3*, both ITV-environment correlations resulted in higher probabilities of Type II error as the correlation between sample size and environment increased. **D)** *Scenario 4* revealed that ITV-environment correlations of 0.9 could only be detected 100% of the times with 75 individuals, whereas correlations of 0.99 could be detected with 25 individuals out of 100.


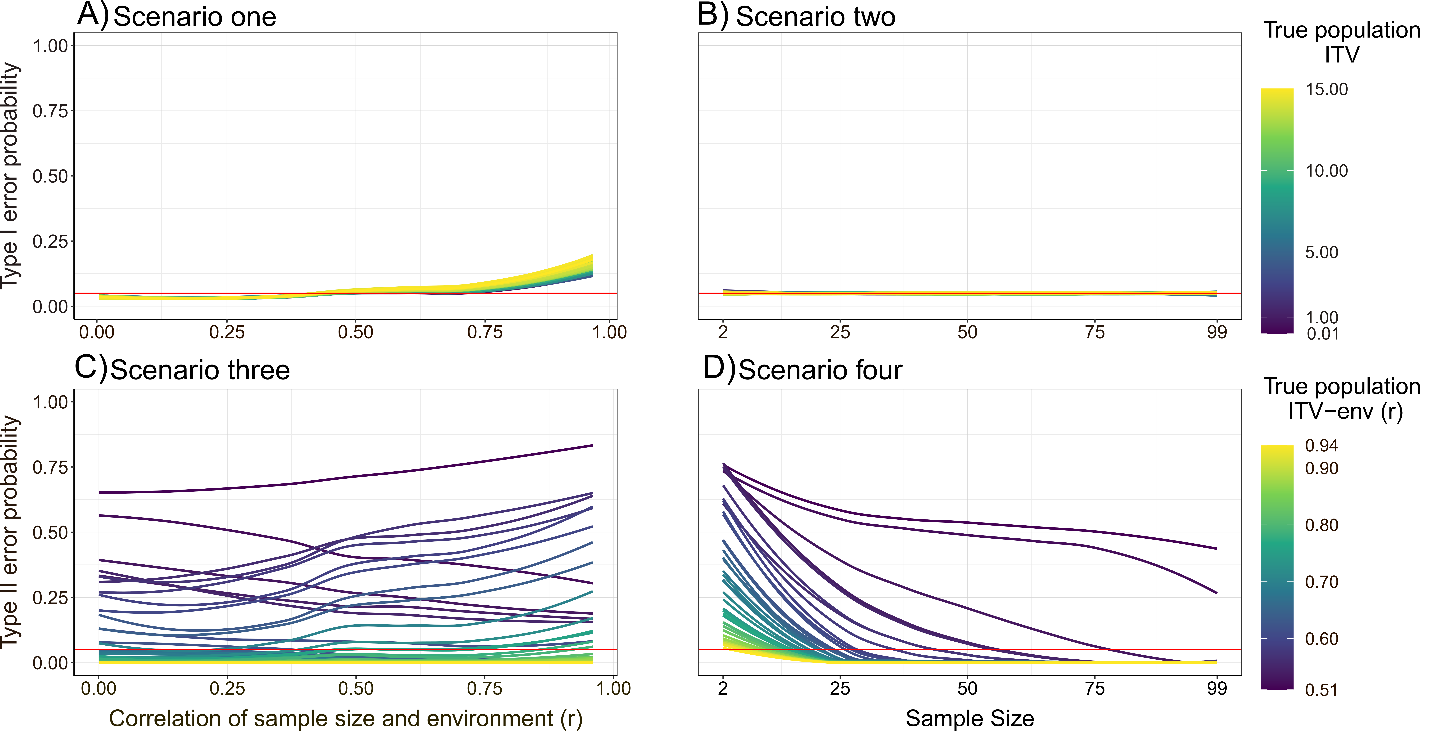


**Figure S6.** *In silico* population simulations for 15 populations. Fifteen populations were evenly distributed across the same environmental gradient ranging from 1 to 10 (1, 1.64, 2.29, 2.93, 3.57, 4.21, 4.86, 5.50, 6.14, 6.79, 7.43, 8.07, 8.71, 9.36, 10). For *Scenarios* which ITV varied across populations, we used population-level ITV values of 0.01, 1.09, 2.18, 3.25, 4.34, 5.42, 6.51, 7.43, 8.50, 9.59, 10.67, 11.75, 12.84, 13.92 and 14.99, and for *Scenarios* which sample size varied along the environment, we used sample sizes of 2, 9, 16, 23, 30, 37, 44, 50, 57, 64, 71, 78, 85, 92 and 99. When ITV and sample size were fixed across populations, 98 values of standard deviation ranged from 0.01 to 15 and sample sizes ranged from 2 to 99. Overall, simulations with 15 populations along the gradient reduced probabilities of both Type I and Type II errors. **A)** In *Scenario 1*, Type I error probability surpasses 5% (red line) only when the correlation between sample size and environment reaches 0.75. **B)** *Scenario 2* mirrored the trends observed for the simulations with 5 and 10 populations, indicating no influence of sample size on Type I error probability when all populations were sampled with an equal number of individuals. When testing Type II error with 15 populations, we had a greater ability to detect ITV-environment relationships (i.e., greater power) than when simulating 5 or 10 populations, but the overall shape of relationships between Type II error probability and the **C)** sample size-environment correlation (*Scenario 3*) and **D)** sample size (*Scenario 4*) were similar to those with fewer populations.


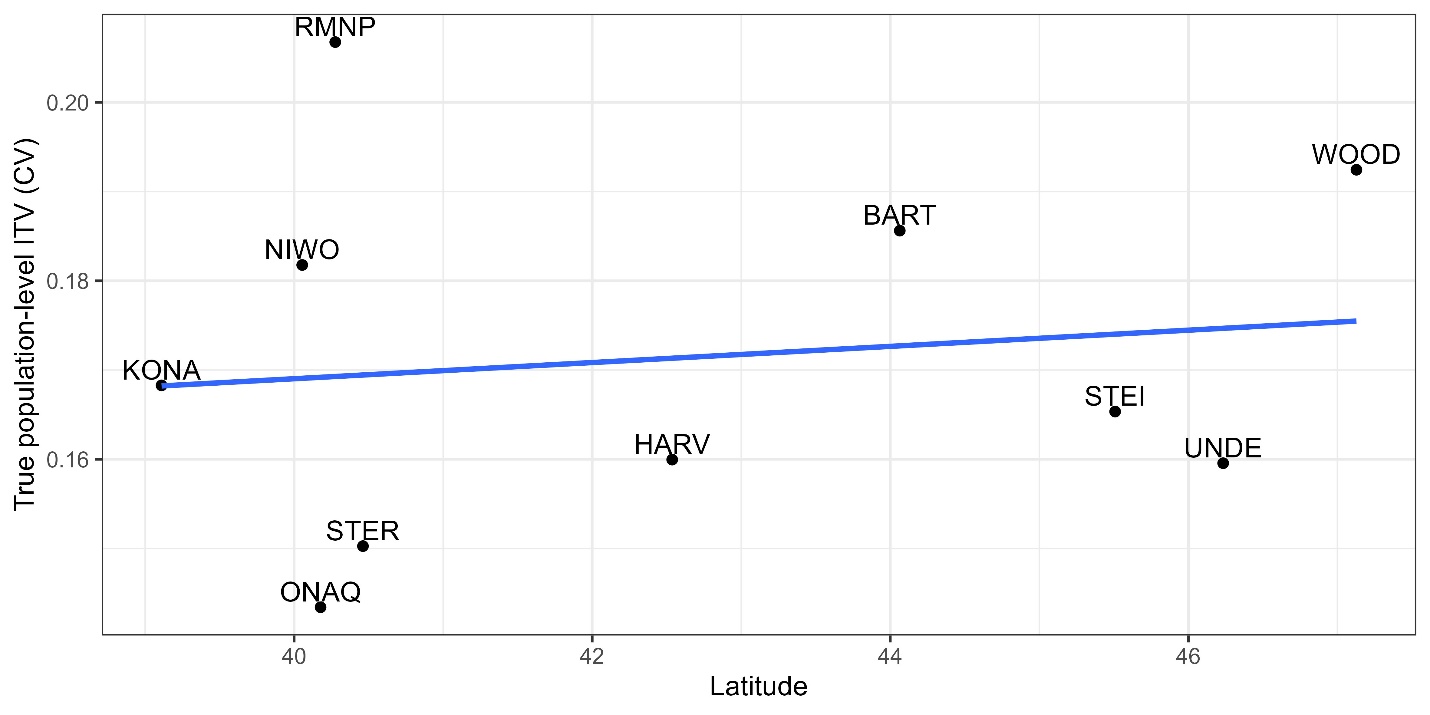


**Figure S7.** Plot showing the non-significant relationship between *P. maniculatus* intraspecific trait variation (ITV) of body weight and latitude in degrees North (*r* = 0.14, *p-value*=0.71). This is the true population-level-ITV relationship used in empirical *Scenarios one and two*. See Table S1 for full site names linked to site acronyms shown here.


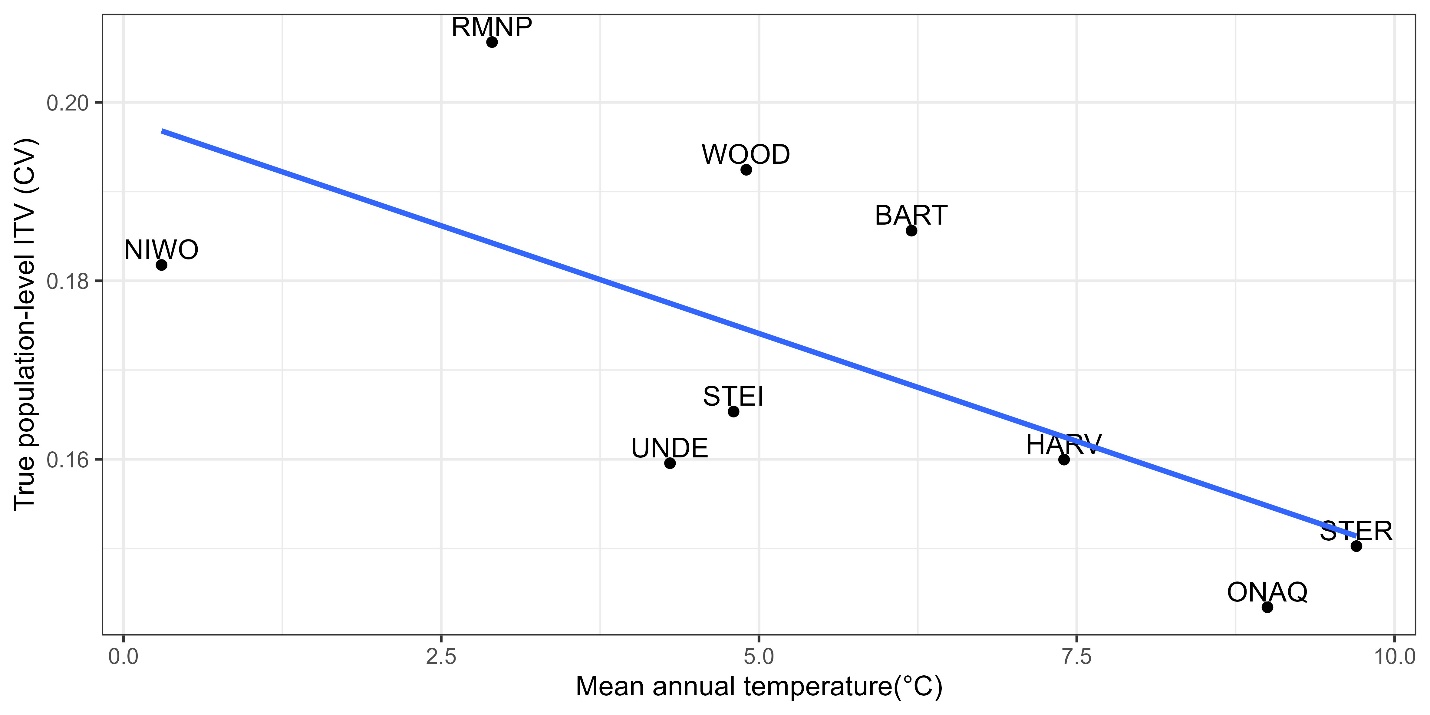


**Figure S8**. Plot showing the significant relationship between *P. maniculatus* intraspecific trait variation (ITV) of body weight and mean annual temperature in degrees Celsius (*r* = 0.68, *p-value*=0.044). This is the true population-level-ITV -environment relationship used in empirical *Scenarios three and four*. See Table S1 for full site names linked to site acronyms shown here.

**Table S1** – Summary of all simulations performed. The theoretical gradient is 1 to 10 (see Figure S1 – S4 for examples).

| Simulation set | Scenario | Estimation | Sample size | Relationship  ITV-environment | Predictor | Response |
| --- | --- | --- | --- | --- | --- | --- |
| 1^st^ set | - | RMSE of population-level ITV | 2 to 99 | - | - | - |
| 2^nd^ set | Scenario 1 | Type I error | Varied | Fixed | Theoretical | In silico population-level ITV (CV) |
|  | Scenario 2 | Type I error | Fixed | Fixed | Theoretical | In silico population-level ITV (CV) |
|  | Scenario 3 | Type II error | Varied | Varied | Theoretical | In silico population-level ITV (CV) |
|  | Scenario 4 | Type II error | Varied | Fixed | Theoretical | In silico population-level ITV (CV) |
| 3^rd^ set | Scenario 1 | Type I error | Varied | Fixed | Latitude | CV of Body weight (g) *P. maniculatus* (10 populations) |
|  | Scenario 2 | Type I error | Fixed | Fixed | Latitude | CV of Body weight (g) *P. maniculatus* (10 populations) |
|  | Scenario 3 | Type II error | Varied | Varied | Temperature (C^o^) | CV of Body weight (g) *P. maniculatus* (9 populations) |
|  | Scenario 4 | Type II error | Varied | Fixed | Temperature (C^o^) | CV of Body weight (g) *P. maniculatus* (9 populations) |

**Table S2 –** Data for NEON sites across the United States and abundance (captures) of *Peromyscus maniculatus* for each site. The dataset was downloaded in March 2023. *Site excluded from empirical analysis in *Scenarios three and four*.

| Site | Site ID | Latitude | Longitude | Temperature ^o^C | Captures | Years of sampling |
| --- | --- | --- | --- | --- | --- | --- |
| Konza Prairie Agroecosystem* | KONA | 39.11045 | -96.6129 | 12.7 | 233 | 2017-2023 |
| Niwot Ridge | NIWO | 40.05425 | -105.582 | 0.3 | 186 | 2015-2022 |
| Onaqui | ONAQ | 40.1776 | -112.452 | 9.0 | 345 | 2014-2023 |
| Rocky Mountains | RMNP | 40.2759 | -105.546 | 2.9 | 183 | 2017-2023 |
| North Sterling | STER | 40.46189 | -103.029 | 9.7 | 269 | 2014-2022 |
| Harvard Forest & Quabbin Watershed | HARV | 42.53691 | -72.1727 | 7.4 | 303 | 2013-2022 |
| Bartlett Experimental Forest | BART | 44.06389 | -71.2874 | 6.2 | 331 | 2014-2022 |
| Steigerwaldt-Chequamegon | STEI | 45.50894 | -89.5864 | 4.8 | 205 | 2015-2023 |
| University of Notre Dame Environmental Research Center | UNDE | 46.23391 | -89.5373 | 4.3 | 415 | 2014-2023 |
| Chase Lake National Wildlife Refuge | WOOD | 47.1282 | -99.2413 | 4.9 | 309 | 2014-2022 |

**Table S3 –** Linear regression results for relationships of latitude and mean annual temperature with CV of P. *maniculatus* body weight. The predictors were tested separately. Latitude analysis was performed using 10 sites and Temperature analysis was conducted using 9 sites.

|  | Slope | SE | t value | *p* | r | Adj. R^2^ | F |
| --- | --- | --- | --- | --- | --- | --- | --- |
| Latitude | 0.091 | 0.235 | 0.386 | 0.710 | 0.14 | -0.105 | 0.149 |
| Temperature | -0.483 | 0.197 | -2.452 | 0.044 | 0.68 | 0.385 | 6.012 |
